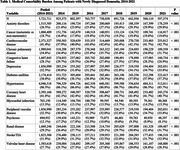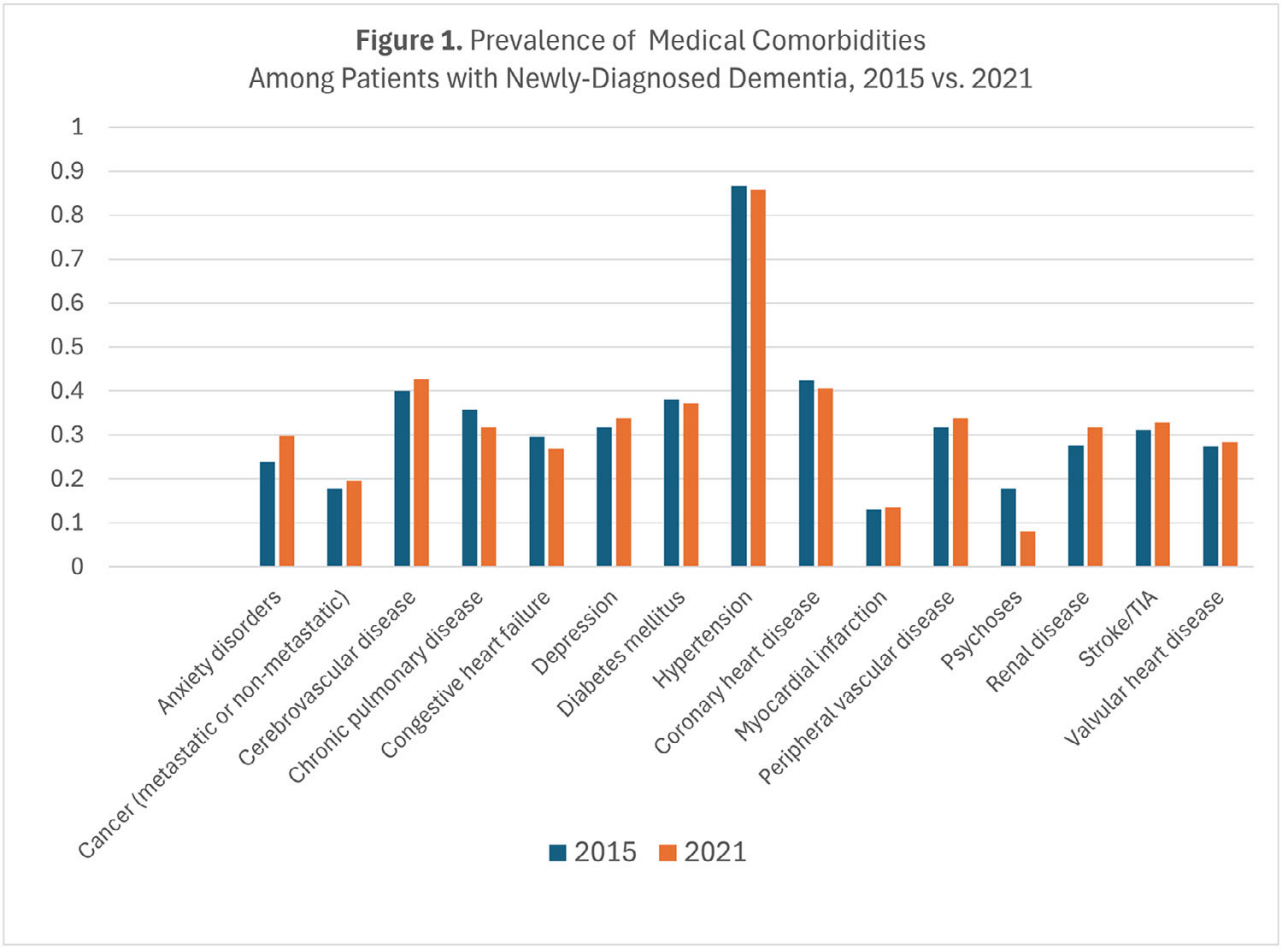# Nationwide Trends in Medical Comorbidity Burden of Patients Newly Diagnosed with Dementia, 2015‐2021

**DOI:** 10.1002/alz.093489

**Published:** 2025-01-09

**Authors:** Jay B Lusk, Cassie B Ford, Beau Blass, Kim G Johnson, Amy G Clark, Samir Soneji, Richard J O'Brien, Emily C O'Brien, Bradley G Hammill

**Affiliations:** ^1^ Duke University, Durham, NC USA

## Abstract

**Background:**

Medical comorbidity burden has a substantial impact on care for patients with dementia and has major impacts on quality of life. No nationwide study has evaluated trends in medical comorbidity burden of patients with a new diagnosis of dementia. We therefore performed a nationwide study of medical claims data to understand the prevalence of comorbid medical conditions at time of dementia diagnosis in real‐world clinical practice.

**Method:**

We studied 100% of nationwide Medicare claims from 2014‐2021 and evaluated the prevalence of medical comorbidities among patients with new diagnoses of dementia. Medical comorbidities were ascertained through searching inpatient and outpatient claims for 1 year prior to dementia diagnosis. We used validated international classification of diseases (ICD) code algorithms to identify both dementia diagnoses and medical comorbidities. We report the number and percentage of patients with each comorbid medical condition.

**Result:**

A total of 5,721,711 patients with incident dementia were included in the study. Anxiety disorders were much more common among patients diagnosed in 2021 (29.8%) compared to patients diagnosed in 2015 (23.8%). Depression was also more common among patients diagnosed in 2021 (33.8%) versus 2015 (31.8%). Hypertension remained very common (85.7% in 2021 vs 86.6% in 2015). There was a major decrease in patients with prior history of psychosis (17.8% in 2015 vs 8.1% in 2021). There were modest increases in the rates of comorbid cardiovascular disease (Table 1). Figure 1 shows the percentage of patients with new diagnosis of dementia with various conditions in 2015 and 2021 respectively.

**Conclusion:**

In a national database of Medicare claims, there were substantial changes from 2015‐2021 in comorbidity burden among patients newly diagnosed with dementia. There was a dramatic decrease in comorbid psychosis which could be attributable to Medicare initiatives to reduce the inappropriate use of antipsychotic medications among patients with dementia. Our results have implications for health system performance and risk adjustment, given an overall increase in comorbidity burden across many disease states from 2015‐2021. Strategies to address rising multimorbidity may be increasingly important for patients with dementia.